# Blocking the PD-1/PD-L1 axis enhanced cisplatin chemotherapy in osteosarcoma in vitro and in vivo

**DOI:** 10.1186/s12199-019-0835-3

**Published:** 2019-12-21

**Authors:** Xiaoqiang Liu, Shaoya He, Huaming Wu, Hui Xie, Tao Zhang, Zhongliang Deng

**Affiliations:** 1 Department of Orthopedic Surgery, The Second Affiliated Hospital of Chongqing Medical University, 74 Linjiang Road, Yuzhong district, Chongqing, 40010 China; 2 Department of Orthopedic Surgery, Sichuan Anyue County People’s Hospital, 68 Wainan Street, Anyue, 642350 China; 3 Department of Gastroenterology, Sichuan Anyue County People’s Hospital, 68 Wainan Street, Anyue, 642350 China; 4 Department of orthopedic Surgery, Guizhou Orthopedics Hospital, 123 Shachong South Road, Guiyang, 550002 China

**Keywords:** Osteosarcoma, PD-L1, Cisplatin, Anti-PD-1 antibody, Treg cell

## Abstract

**Background:**

The blocking of the programmed cell death protein (PD-1)/programmed death-ligand 1 (PD-L1) axis has been found to have an anticancer activity against various types of cancer by enhancing T cell immunity, while there are no studies linking the PD-1/PD-L1 axis to chemotherapy drugs in osteosarcoma (OS). The present study aimed to investigate the effects of blocking PD-1/PD-L1 axis on the cisplatin chemotherapy in OS in vitro and in vivo.

**Methods:**

Reverse transcription-quantitative polymerase chain reaction (RT-qPCR) was applied to detect PD-L1 mRNA in OS tissues. Cell proliferation and apoptosis were measured by Cell Counting Kit-8 (CCK-8) and flow cytometry assays, respectively. In vivo, the syngeneic mice were treated with cisplatin and anti-PD-1 antibody alone or jointly.

**Results:**

In this study, it revealed that PD-L1 mRNA was highly expressed in OS tissues. Further inhibitory evaluation showed that the K7M2-LV cells (PD-L1 overexpression) co-cultured with PD-1^+^ lymphocytes could promote K7M2 cell proliferation. Meanwhile, the combination of anti-PD-1 antibody and cisplatin significantly decreased the proliferation and increased the apoptosis of K7M2 cells in a co-culture system. In vivo, the combination of anti-PD-1 antibody and cisplatin significantly inhibited tumor growth, while the mechanisms did not involve regulatory T cells.

**Conclusion:**

The present data suggested that the blocking of PD-1/PD-L1 axis had a positive prognostic value, which can enhance the chemotherapeutic effect of cisplatin in OS. These findings provide a rationale for utilizing PD1/PD-L1 blocking antibodies as a single agent to cure refractory OS in patients receiving cisplatin treatment.

## Introduction

OS is the fifth most common type of pediatric cancer worldwide, ranking third in pediatric cancer-related mortality [1]. Despite major advances in diagnosis and clinical treatment, the prognosis for patients with OS remains poor, with the 5-year survival rate at 30–40% [2], partly due to chemoresistance. Therefore, improving the sensitivity of chemotherapy drugs to control OS is urgently required.

Tumorectomy followed by local radiotherapy and systemic chemotherapy is the standard therapy for advanced OS [3]. The current national and international cooperative trial for patients with newly diagnosed OS is built on the backbone of cisplatin, doxorubicin, and methotrexate [4]. Now believe that the tumor chemotherapy effect in addition to the sensitivity to chemotherapy drugs related to tumor cells and also related to the body’s immune status, immune suppression, or immune escape is one of the main reasons for the effect of chemotherapy [5]. Immune suppression driven by PD-1/PD-L1 inhibits the functions of T cells in a broad range of cancer types, including OS [6]. Studies have shown that endogenous antitumor agents, such as interferon –γ, could induce a high expression of PD-L1 in certain types of cancer to reduce the chemotherapeutic effect; therefore, blocking the PD-1/PD-L1 pathway should increase the effect of chemotherapy [7]. However, there are no studies on the association between markers of immune response and chemotherapy drugs in OS.

The aim of this study was to evaluate whether the blocking the PD-1/PD-L1 axis has a positive prognostic value and whether it can enhance the chemotherapeutic effect of cisplatin in human OS. In the end, it revealed that PD-L1 mRNA was significantly upregulated in OS tissues, and the interactions of PD-1 and PD-L1 promoted K7M2 cell proliferation. Meanwhile, the blocking of PD-1/PD-L1 axis by anti-PD-1 antibody enhanced the anti-tumor efficacy of cisplatin in OS.

## Materials and methods

### Animals

Thirty-two male Balb/c nude mice (16–18 g, 4–5 weeks old) were purchased from the animal center of the West China Medical College of Sichuan University, Chengdu, China. All animals were housed with free access to food and water under specific pathogen-free conditions. Following the mice were acclimated for 1 week prior to study initiation. The duration of the experiment was 27 days. All experiments were performed in accordance with the National Institutes of Health Guide for the Care and Use of Laboratory Animals (NIH Publications No. 8023, revised 1978) and were approved by the Ethical Committee of the west China Hospital of Sichuan University (Chengdu, China). The associated permit numbers is 2019119A.

### Tissue samples

A total of 6 pairs of human OS and corresponding OS-adjacent tissues were obtained from patients who underwent surgery at the Orthopedic Surgery Department of the Second Affiliated Hospital, Chongqing Medical University (Chongqing, China), between April 2016 and April 2017. No patient had previously received preoperative treatment, and this was the first surgical resection for all patients. The study protocol was approved by the Ethics Review Board at the Second Affiliated Hospital, Chongqing Medical University. Written informed consent was obtained from each patient.

### Immunohistochemistry (IHC)

The PD-L1 expression in OS tissues and cells was analyzed by IHC. Briefly, tissues were deparaffinized, rehydrated, and subjected to antigen retrieval by 3% hydrogen peroxide. The sections were then incubated with a primary PD-L1 antibody (Abcam, Cambridge, USA) overnight at 4 °C and incubated with a secondary antibody (OriGene Technologies, Inc., Beijing, China) for 30 min at 37 °C. The reaction was visualized using 3,3′-diaminobenzidine (DAB; OriGene Technologies, Inc.) and counterstained with hematoxylin. The OS cells were not deparaffinized or rehydrated. Brown or yellow granules in the cytoplasm or nucleus were considered positive immune staining. A Nikon imaging system was used for image collection. The positive area ratio of each field was analyzed using Image-ProPlus software.

### RNA isolation and reverse transcription-quantitative polymerase chain reaction

Total RNA was extracted from tissues using TRIzol reagent (Invitrogen; Thermo Fisher Scientific, Inc., Waltham, MA, USA). Reverse transcription was performed using the PrimeScript™RT reagent Kit (Takara Biotechnology Co., Ltd., Dalian, China) to synthesize cDNA. Subsequently, the primer and SYBR Premix Ex Taq II (Takara Biotechnology Co., Ltd.) were used to detect the expression of PD-1 and PD-L1. The mRNA content was normalized to the housekeeping gene β-actin. The specific primer sequences were as follows: PD-L1, 5′-AGAACTACCTCTGCACATCCTCCAA-3′ forward and 5′-CCATTCCTTCCTCTTGTCACGCTCAG-3′ reverse; β-actin, 5′-GAAGATCAAGATCATTGCTCCT-3′ forward and 5′-TACTCCTGCTTGCTGATCCA-3′ reverse.

### Lentivirus preparation

Lentiviral particles in the absence or presence of the PD-L1 gene were obtained from Shanghai GeneChem Co., Ltd. (Shanghai, China). The viruses were used to infect cells in the presence of polybrene and enhanced infection solution (Shanghai GeneChem Co., Ltd.). To obtain stable PD-L1 clones from the K7M2 cell line, the green fluorescent expression was observed in infected cells under a fluorescent microscope.

### Lymphocyte sorting

The CD4^+^PD-1^+^ and CD4^+^PD-1^−^ lymphocytes were sorted by flow cytometry. Briefly, K7M2 cells treated with lentiviral transfection were inoculated in 16 Balb/c nude mice. Once the tumor diameter reached 1–1.5 cm, the mice were euthanized by CO_2_ inhalation, and peripheral blood (*n* = 6 mice/group) was collected for lymphocyte separation. Next, the collected lymphocytes were incubated with anti-mouse CD279 (PD-1), anti-mouse CD8, and anti-mouse CD4 (BioLegend, CA, USA), and CD4^+^PD-1^+^ and CD4^+^PD-1^−^ lymphocytes were sorted using a FACSCalibur™ Flow Cytometer (BD Biosciences, San Jose, CA, USA).

### Cell viability by CCK-8 assay

K7M2 cells (2 × 10^5^/ml) treated with lentiviral transfection were suspended and cultured in a 96-well plate. After 12 h, the CD4^+^PD-1^+^ and CD4^+^PD-1^−^ lymphocytes were seeded at a density of 2 × 10^6^/ml in a 96-well plate and co-cultured with K7M2 cells for 24, 48, and 72 h. Cells were then cultured in 10% CCK-8 (Biosharp, Anhui, China) diluted in fresh medium and were further incubated for 1 h. Cell viability was measured at 450 nm on a microplate reader (Thermo Fisher Scientific, Inc., MA, USA).

### Apoptosis analysis

K7M2 cells treated with lentiviral transfection were suspended and cultured in a 6-well plate. After 12 h, the CD4^+^PD-1^+^ lymphocyte were co-cultured with K7M2 cells. The cells were then divided into four groups, including the control, anti-PD-1 antibody (Anti-PD-1), cisplatin (Cis; Sigma-Aldrich; Merck KGaA, Darmstadt, Germany), and anti-PD-1 antibody combined with cisplatin (anti-PD-1+ Cis) groups. These co-cultured cells were collected and resuspended in 500 μl binding buffer for apoptosis detection following treatment for 24 h. Next, annexin V-fluorescein isothiocyanate (FITC; 5 μl) and propidium iodide (PI; 5 μl) were added to the cell suspension and incubated for 10 min at room temperature in the dark; they were then detected within 1 h using FACSCalibur™ Flow Cytometer.

### Syngeneic mice and regulatory T (Treg) cell assays

Balb/c nude mice (*n* = 16) were subcutaneously injected with 200 μl sterile PBS containing a K7M2 cell suspension at a density of 1 × 10^6^/ml. Once the tumor diameter reached 4–6 mm, the largest volume/diameter of the tumor was 50 mm^3^/6 mm, the mice were divided into 4 groups, including the control (PBS; 100ul/rat), anti-PD-1 antibody (1 mg/kg), cisplatin (5 mg/kg), and anti-PD-1 antibody combined with cisplatin (*n* = 4) groups. The drugs were administered by an intraperitoneal injection, once every 3 days, 5 times in total [8, 9]. Once treatment was complete, mice were sacrificed by CO_2_ inhalation and tumors measured. To alleviate the pain of the mice, the procedure was terminated on day 27 when maximum tumor diameters and volumes of control group reached 15 mm and 1300 mm^3^, respectively. The spleen was collected to prepare single cell suspension (*n* = 4 mice/group). Anti-mouse CD25, CD4, and forkhead box P3 (Foxp3) antibodies (BioLegend, Inc., San Diego, CA, USA) were then added to the cell suspension, and Treg cells were sorted using a FACSCalibur™ Flow Cytometer.

### Statistical analysis

Statistical analysis was performed using SPSS 17.0 (SPSS, Inc., Chicago, IL, USA). The data are expressed as the mean ± standard deviation and derived from three independent experiments. Differences among multiple groups were compared by one-way analysis of variance (ANOVA) with Dunnett’s post hoc test or two-way ANOVA with Bonferroni’s post hoc test, and differences between two groups were compared by the Student’s *t* test. *P* values < 0.05 were considered to indicate statistically significant differences, and < 0.01 were considered highly significant.

## Results

### The expression of PD-L1 increased in OS tissues

**Fig. 1:**
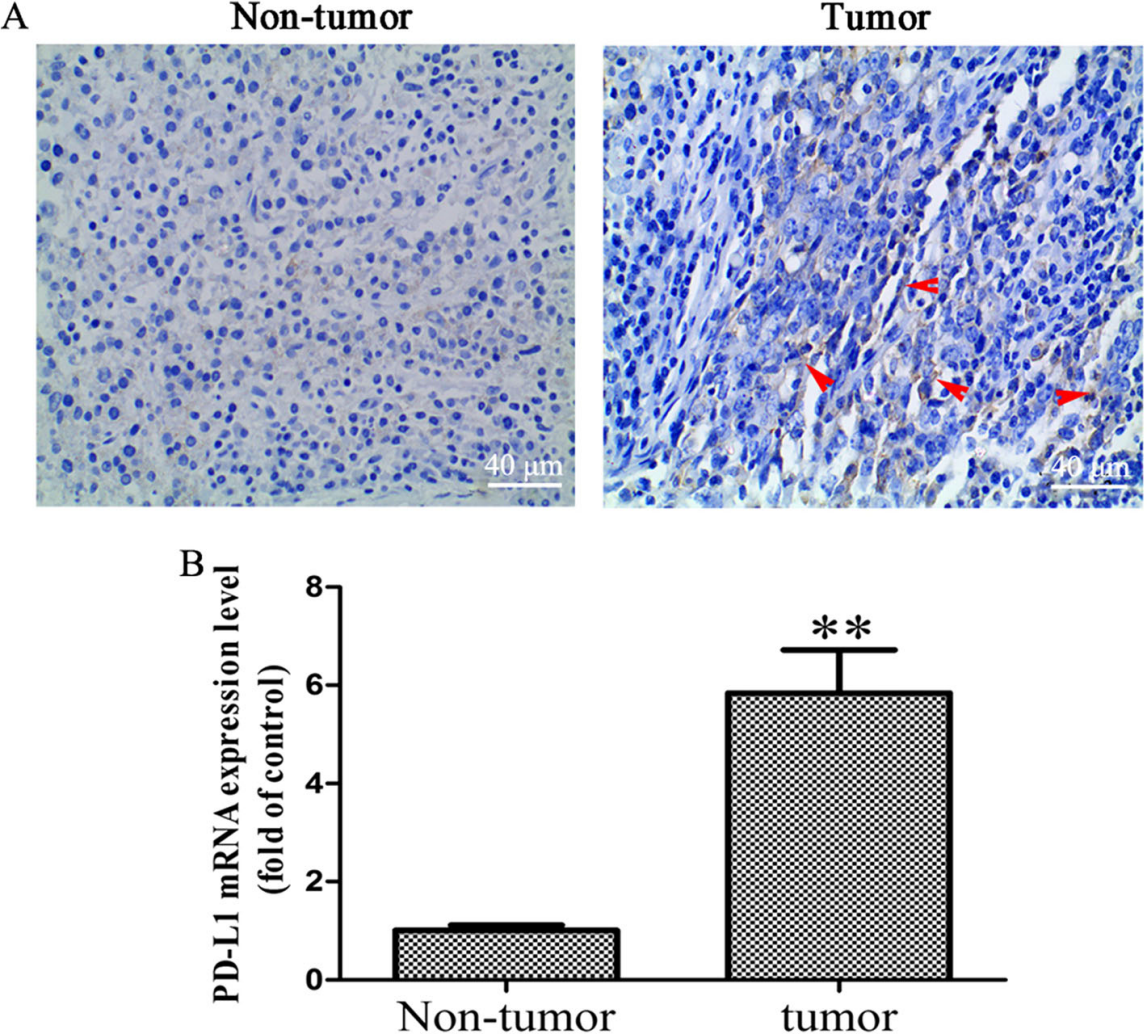
Increased PD-L1 expression level in OS tissues. **a** Immunohistochemical assay was performed to detect the expression of PD-L1 in OS and OS-adjacent tissues (× 400 magnification), the red arrow represents positive PD-L1 staining. **b** RT-qPCR was performed to detected the mRNA level of PD-L1 in OS and OS-adjacent tissues (*n* = 6). The results were presented as the mean ± standard deviation. **P < 0.01. PD-L1, programmed death-ligand 1; OS, osteosarcoma; RT-qPCR, reverse transcription-quantitative polymerase chain reaction

To determine whether PD-L1 blockade should be pursued as a treatment for OS patients, the expression of PD-L1 was detected in human OS tissues. As shown in Fig. 1a, the results showed that the PD-L1 protein in OS tissues was slightly higher than that in OS-adjacent tissues. However, the expression of PD-L1 mRNA in OS tissues was significantly increased, as compared with OS-adjacent tissues (Fig. 1b; *P* < 0.01). Koirala et al. reported that only 7% of the OS tissue samples stained positive for PD-L1, with the majority of positive tumors demonstrating ≤ 25% staining, while the expression of PD-L1 mRNA with high expression [10]. Our results were consistent with Koirala P’s study.

### PD-L1 gene was overexpressed in K7M2-LV cells

**Fig. 2:**
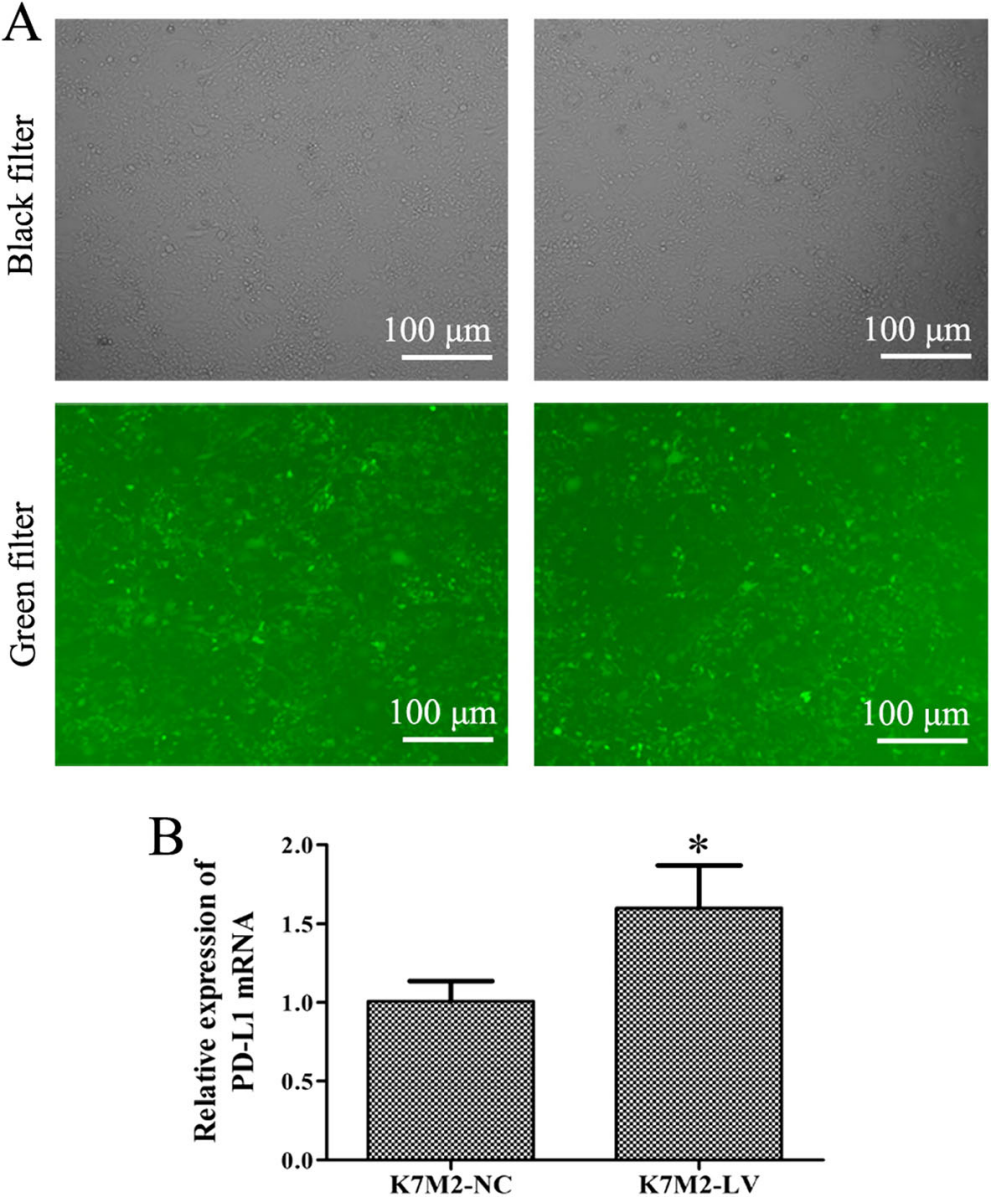
Detection of lentivirus transfection efficiency in K7M2 cells. K7M2 cells were transfected with the lentivirus carried both of GFP or PD-L1 gene for 72 h. **a** Images of K7M2 cells were captured using a fluorescent microscope following transfection with lentivirus (× 100 magnification). **b** RT-qPCR assay was applied to detect the PD-L1 mRNA expression level. *P < 0.05. The experiment was repeated three times, and results are presented as the mean ± standard deviation. GFP, green fluorescent protein; PD-L1, programmed death-ligand 1

The lentivirus carried both of PD-L1 gene and green fluorescent protein (GFP), and the GFP expression could reveal transfection efficiency of K7M2 cells. Multiplicity of infection (MOI) is the average number of viruses per cell. The present data showed that the GFP expression in K7M2 cells was clearly increased following viral infection (Fig. 2a). The MOI was 20 and the infection efficiency up to ≤ 80%. K7M2-LV cells represent the K7M2 cells transfected with lentivirus of PD-L1 presence, while K7M2-NC cells those transfected with lentivirus of PD-L1 absence. The RT-qPCR results showed that the PD-L1 mRNA was significantly increased in K7M2-LV cells compared that in K7M2-NC cells (Fig. 2b). These results indicated that a cell model of PD-L1 gene overexpression was successfully constructed.

### CD4^+^PD-1^+^ lymphocytes co-cultured with K7M2-LV cells promoted K7M2 cell proliferation

The expression of PD-1 ligands by tumors and their interaction with PD-1-expressing T cells in the tumor microenvironment can result in tolerance [11].

**Fig. 3:**
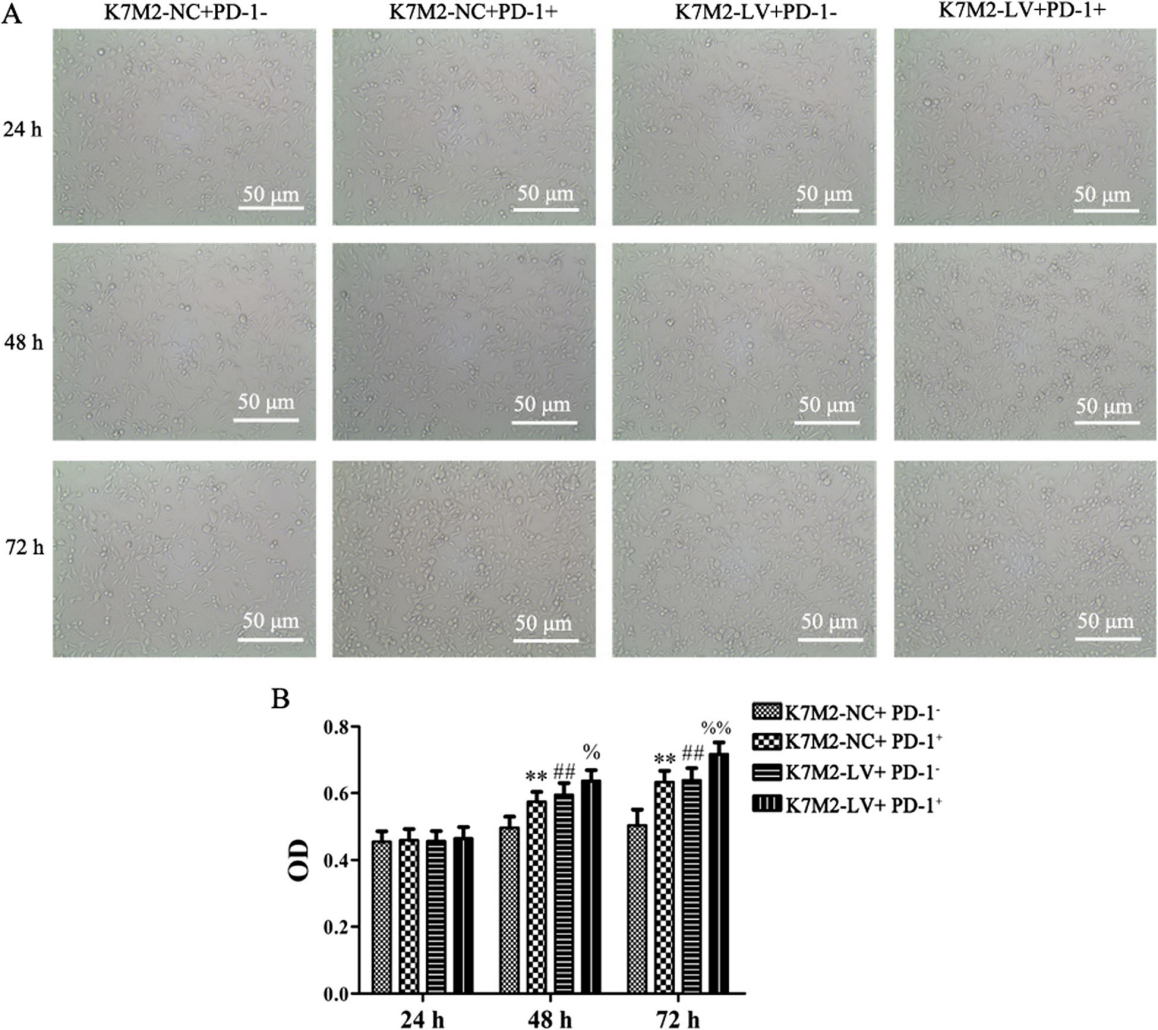
Interaction between PD-L1 and PD-1 can promote K7M2 cell proliferation. K7M2-LV or K7M2-NC cells were co-cultured with CD4+PD-1+ or CD4+PD-1− lymphocytes for 24, 48, and 72 h. **a** Cell morphology was observed under microscopes (× 200 magnification). **b** Relative cell viability was determined using a CCK-8 assay. The experiment was repeated three times, and results are presented as the mean ± standard deviation. **P < 0.01 and ##*P* < 0.01 vs the K7M2-NC + PD-1− group; %*P* < 0.05 and %%*P* < 0.01 vs the K7M2-LV + PD-1− group. PD-L1, programmed death-ligand 1; PD-1, programmed cell death protein

The present study examined the viability of K7M2 cells in a lymphocyte (PD-1^+^/^−^) co-culture system with K7M2-LV (or K7M2-NC) cells. As shown in Fig. 3, the K7M2-NC cell viability was significantly increased following co-culture with PD-1^+^ lymphocytes for 48 and 72 h, as compared with that in the K7M2-NC+ PD-1^−^ group (*P* < 0.01). The K7M2-LV cell viability was significantly increased following co-culture with PD-1^+^ lymphocytes for 48 and 72 h, as compared with that in the K7M2-LV+ PD-1^−^ group (*P* < 0.01). Finally, the K7M2-LV cell viability was significantly higher than that of K7M2-NC (*P* < 0.01). These data indicated that the overexpression of PD-L1 may promote the OS malignancy and that interaction between PD-L1 and PD-1 could result in tumor immune escape and promote K7M2 cell proliferation.

### Anti-PD-1 antibody combined with cisplatin decreased the proliferation and increased the apoptosis of K7M2 cells

**Fig. 4:**
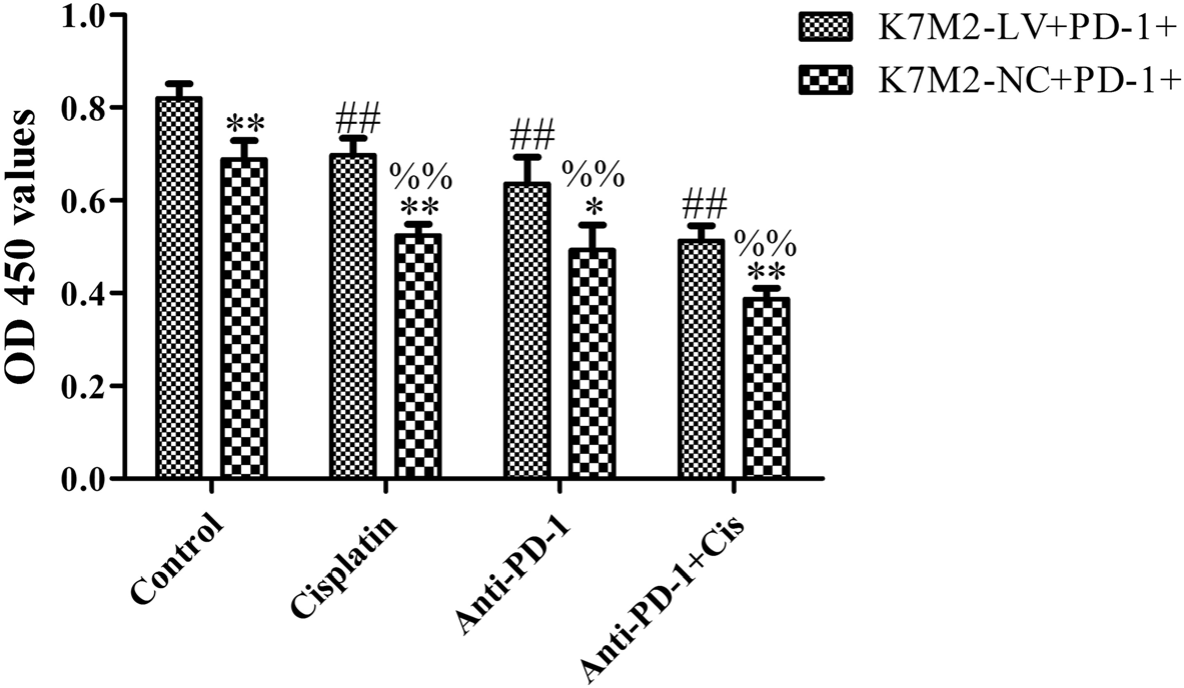
Anti-PD-1 antibody synergized with cisplatin to inhibit cell proliferation. K7M2-LV or K7M2-NC cells were co-cultured with PD-1+ lymphocytes, followed by incubation with anti-PD-1 antibody (1 μg/ml) and cisplatin (2 μg/ml) alone or jointly for 48 h. Cell viability was determined using a CCK-8 assay. The experiment was repeated three times, and results are presented as the mean ± standard deviation. *P < 0.05 and **P < 0.01 vs. the K7M2-LV + PD-1+ group; ##*P* < 0.01 vs. the control group (K7M2-LV + PD-1+); %%*P* < 0.01 vs. the control group (K7M2-NC + PD-1+). PD-1, programmed cell death protein

**Fig. 5:**
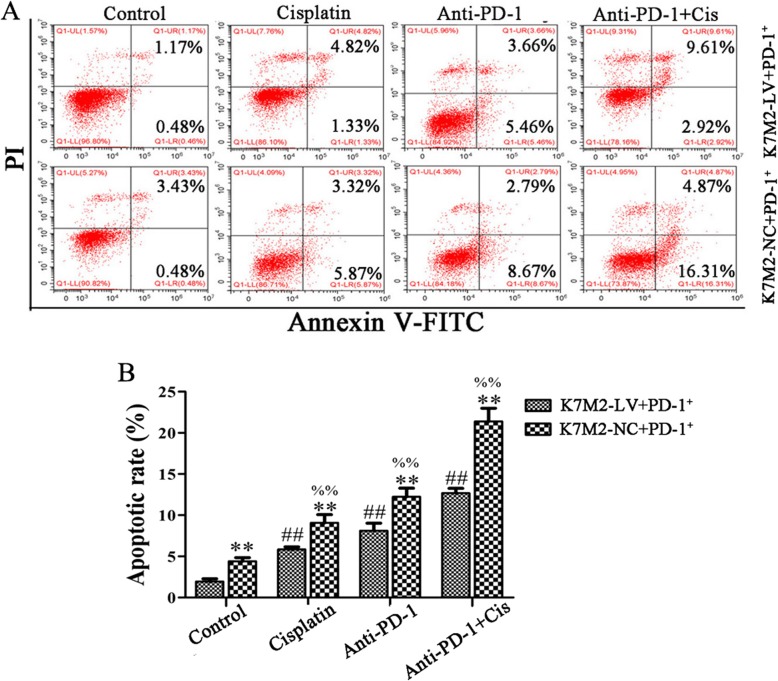
Anti-PD-1 antibody synergized with cisplatin to promote cell apoptosis. K7M2-LV or K7M2-NC cells were co-cultured with PD-1+ lymphocytes, followed by incubation with anti-PD-1 antibody (1 μg/ml) and cisplatin (2 μg/ml) alone or jointly for 48 h. **a** Apoptotic cells were detected by flow cytometry. **b** The apoptotic rate of K7M2 cells was statistically analyzed. The experiment was repeated three times, and results are presented as the mean ± standard deviation. **P < 0.01 vs. the K7M2-LV + PD-1+ group; ##*P* < 0.01 vs. the control group (K7M2-LV + PD-1+); %%*P* < 0.01 vs. the control group (K7M2-NC + PD-1+). PD-1, programmed cell death protein

The main aim of the present study was to enhance the sensitivity of immune escape tumor cells to cisplatin chemotherapy. As shown in Figs. 4 and 5, the cell apoptotic rate was significantly increased following treatment with anti-PD-1 antibody combined with cisplatin, as compared to that following treatment with anti-PD-1 antibody or cisplatin alone in the K7M2-NC + PD-1^+^ and K7M2-LV + PD-1^+^ groups, respectively (*P* < 0.01), while the change of cell proliferation is opposite to that of apoptosis. These results suggested that PD-1 antibody can synergize with cisplatin in OS. The cell apoptotic rate was significantly increased in the K7M2-NC + PD-1^+^ group following treatment with anti-PD-1 antibody and cisplatin alone or jointly, as compared to the K7M2-LV + PD-1^+^ group (*P* < 0.01), while the change of cell proliferation is opposite to that of apoptosis, indicating that the interaction between PD-L1 and PD-1 could result in tumor immune escape and inhibit apoptosis in K7M2 cells.

### Anti-PD-1 antibody combined with cisplatin inhibited the protein expression of PD-L1 of K7M2 cells

**Fig. 6:**
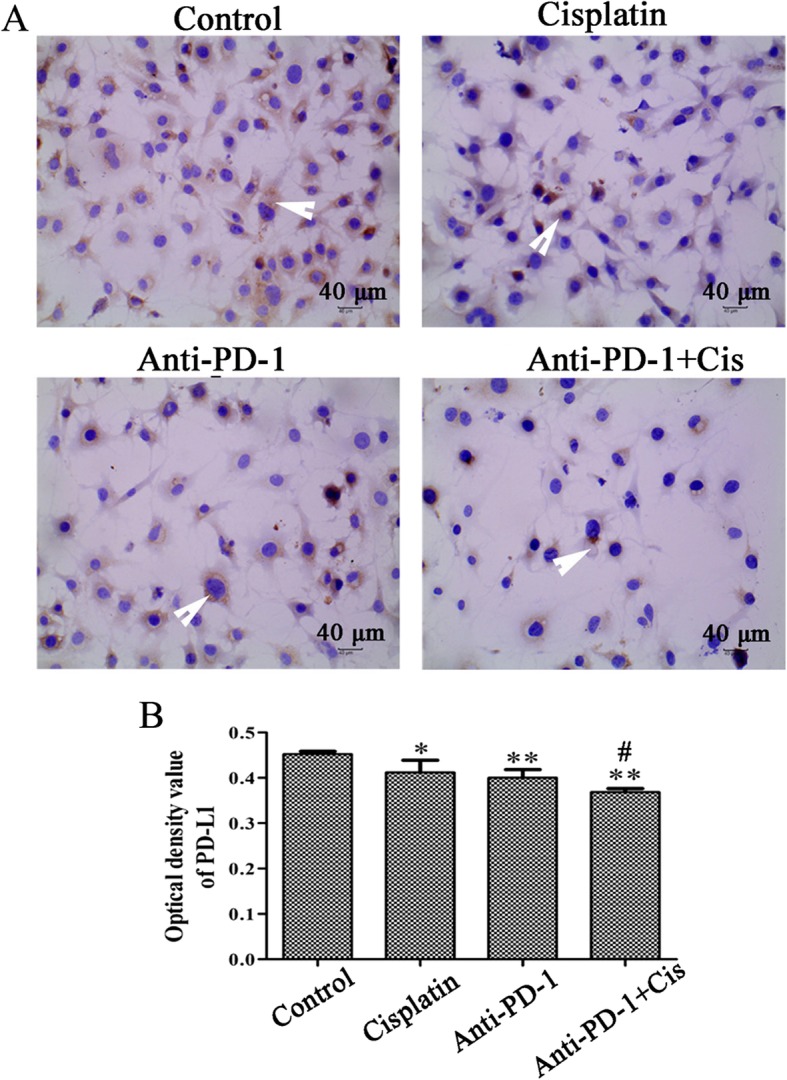
Increase of PD-L1 expression level in K7M2 cells. K7M2 cells were incubated with anti-PD-1 antibody (1 μg/ml) and cisplatin (2 μg/ml) alone or jointly for 48 h. **a** Immunohistochemical assay was performed to detect the expression of PD-L1 in K7M2 cell (× 400 magnification); the white arrow represents positive PD-L1 staining. **b** The optical density value of PD-L1 was statistically analyzed. The experiment was repeated three times. Results are presented as the mean ± standard deviation. **P < 0.01 and *P < 0.05 vs control group; #*P* < 0.05 vs cisplatin group. PD-L1, programmed death-ligand 1; PD-1, programmed cell death protein

To further explore the anti-tumor mechanism of PD-1 antibody sensitized cisplatin, the PD-L1 protein expression was detected in K7M2 cells. The positive products of PD-L1 were yellow or brown, mainly distributed in the cytoplasm. Compared with the control group, the expression of the PD-L1 protein in K7M2 cells was significantly decreased in cisplatin, anti-PD-1 antibody, and combined groups (*P* < 0.05). The expression of the PD-L1 protein in the combined group was also significantly lower than that in the cisplatin group (Fig. 6; *P* < 0.05).

### Tumor growth delay by cisplatin and anti-PD-1 antibody does not involve Treg cells

**Fig. 7:**
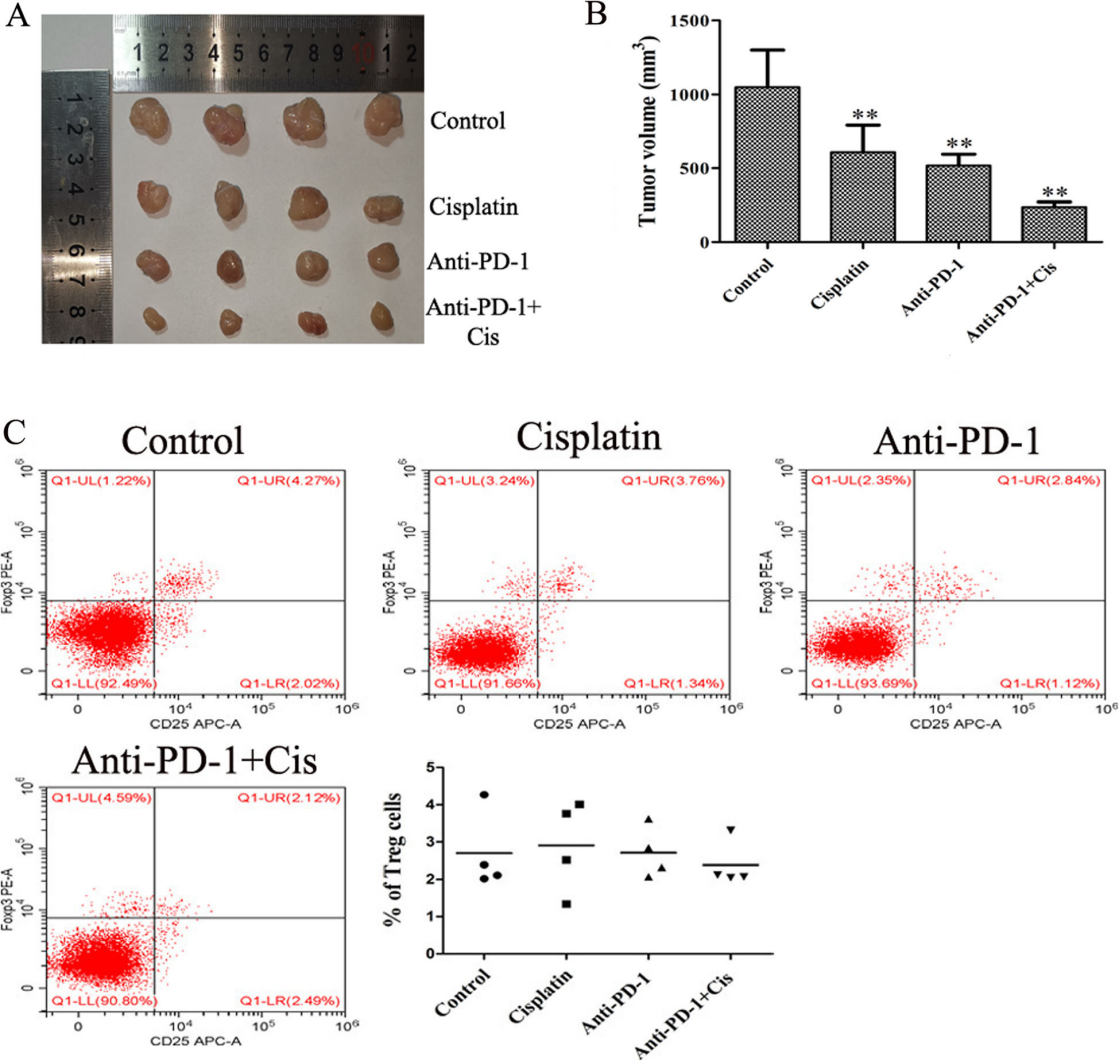
Anti-PD-1 antibody combined with cisplatin inhibited tumor growth without reducing the number of Treg cells. Tumors were harvested from a subset of K7M2 tumor-bearing mice on day 27 following tumor cell injection. Ten days after inoculation, mice were treated intraperitoneally with PBS (100 ul/rat), 5 mg/kg cisplatin, 1 mg/kg anti-PD-1 antibody, and cisplatin (5 mg/kg) plus anti-PD-1 antibody (1 mg/kg), once every 3 days, 5 times in total. **a**, **b** Mean tumor volumes in each group (*n* = 4). **c** Treg cells were analyzed by flow cytometry (*n* = 4/group). The experiment was repeated three times. Results are presented as the mean ± standard deviation. **P < 0.01 vs. the control group. PD-1, programmed cell death protein; Treg, regulatory T

To elucidate the mechanisms through which cisplatin and PD-L1/PD-1 blockade induces a tumor growth delay in K7M2 tumors in vivo, the mice were treated with cisplatin and anti-PD-1 antibody alone or jointly, and there is no mice died in each group during the course of the experiment. As shown in Fig. 7a, b, the tumor volume was significantly decreased following treatment with anti-PD-1 antibody combined with cisplatin for 15 days (*P* < 0.01). The maximum tumor diameter was 15.04 mm, and the maximum volume was 1223.617 mm^3^. However, no obvious change was observed in the percentage of Treg cells in each group (Fig. 6c), indicating that the anti-tumor mechanism of anti-PD-1 antibody and cisplatin were independent of Treg cell consumption.

## Discussion

The activation of PD-1/PD-L1 axis plays an important role in the immunosuppressive tumor microenvironment, protecting tumor cells escape and metastasis from antitumor immunity [12]. PD-L1 has been associated with various human cancers including colorectal, pancreatic, and prostate cancer [13]. Several studies have identified PD-L1 as a potential prognostic biomarker and therapeutic target [14, 15]. The present data showed that the expression of the PD-L1 gene in OS tissues was higher than that in OS-adjacent tissues. However, due to the difficulty of sample collection, there are no clinical samples in this study, which is a limitation of our study. Fortunately, other large sample studies have demonstrated increased PD-L1 expression in OS tissues and can be an independent risk factor for overall survival in OS patients [6, 16], indicating the PD-L1 may be a prognostic biomarker in OS.

PD-L1, a member of the B7 family, negatively regulates T cell immune responses by binding to PD-1, an immunosuppressive receptor of the CD28 family [17]. Iwai et al. [18] reported that the blockade of PD-1 inhibits hematogenous spread of poorly immunogenic tumor cells by enhanced recruitment of effector T cells. Okudaira et al. [19] reported that the blocking of B7-H1 (PD-L1) or B7-DC (PD-L2) induces an anti-tumor effect in a mouse pancreatic cancer model. Thus, to further study the role of immune escape in OS, the lentiviral particles of PD-L1 gene absence or presence was used to infect K7M2 cells and then co-cultured with CD4^+^PD-1^+^ or CD4 + PD-1^−^ lymphocytes in vitro. The results revealed that the CD4^+^PD-1^+^ lymphocytes co-cultured with PD-L1 overexpressed K7M2 cells could promote cell proliferation, indicating that the PD-1 combined with PD-L1 in K7M2 cells could lead an immunosuppressive tumor microenvironment and promote the survival of OS. Therefore, blocking the PD-1/PD-L1 axis may be an effective treatment in OS.

Currently, most OS patients receive adjuvant chemotherapy after surgical removal of all detectable diseases [20]. Cisplatin, a cell-cycle nonspecific drug that inhibits DNA replication, has been widely used in postoperative chemotherapy for various cancers, including OS [21]. Unfortunately, resistance to cisplatin limited the chemotherapeutic efficacy in OS [22]. Therefore, enhancing the sensitivity of OS to cisplatin is the key to treatment. The survival of tumors in several cases is assisted by checkpoint immunomodulation to maintain the imbalance between immune surveillance and cancer cell proliferation [23]. Immune checkpoint blockade is considered as a new paradigm for the treatment of advanced cancer [24]. In recent times, more than four checkpoint antibody inhibitors targeting PD-1, PDL-1, and CTLA-4 have been commercialized, and these checkpoint blockers are rapidly becoming a promising cancer treatment with significant anti-tumor responses and limited side effects [23]. In addition, PD-1 inhibitors are being combined with anti-tumor drugs, such as cisplatin, to achieve long-lasting synergistic anticancer effects [25]. Tran et al [26] reported that the PD-1/PD-L1 inhibition by anti-PD-L1 antibody could synergize cisplatin enhance the anti-tumor efficacy in head and neck squamous cell carcinoma. In the present study, it revealed that the blocking of PD-1/PD-L1 axis was significantly enhanced the anti-tumor efficacy of cisplatin in OS, which is reflected in decreased cell proliferation, increased cell apoptosis, and decreased tumor volume. The main mechanism of tumor resistance is the acquisition by tumor cells of the immune evasion capability, through the induction of cells with immune suppressive properties, such as Treg cells [27]. However, the present data showed no marked change in the number of Treg cells in each group, indicating that the anti-tumor mechanism of anti-PD-1 antibody combined with cisplatin were independent of Treg cell consumption.

## Conclusion

In conclusion, the present study showed that PD-L1 mRNA with high expression was measured in OS tissues and demonstrated that the overexpression of PD-L1 could promote proliferation and immune escape in OS K7M2 cells. It was also showed that anti-PD-1 antibody could enhance the anti-tumor effect of cisplatin through decreasing proliferation, increasing apoptosis, and inhibiting tumor growth, without reducing the number of Treg cells. These findings provide a rationale for utilizing PD-1/PD-L1 blocking antibodies as a single agent to cure refractory OS in patients receiving cisplatin treatment. However, the anti-OS mechanisms of anti-PD-1 antibody combined with cisplatin remain to be further elucidated.

## Data Availability

The datasets used or analyzed during the current study are available from the corresponding author on reasonable request.

## Abbreviations

ANOVA: One-way analysis of variance
CCK-8: Cell Counting Kit-8
DAB: 3,3′-Diaminobenzidine
FITC: Fluorescein isothiocyanate
Foxp3: Forkhead box P3
GFP: Green fluorescent protein
IHC: Immunohistochemistry
MOI: Multiplicity of infection
OS: Osteosarcoma
PD-1: Programmed cell death protein 1
PD-L1: Programmed death-ligand 1
PI: Propidium iodide
RT-qPCR: Reverse transcription-quantitative polymerase chain reaction
Treg: Regulatory T
